# Enhanced effects of biotic interactions on predicting multispecies spatial distribution of submerged macrophytes after eutrophication

**DOI:** 10.1002/ece3.3294

**Published:** 2017-08-22

**Authors:** Kun Song, Yichong Cui, Xijin Zhang, Yingji Pan, Junli Xu, Kaiqin Xu, Liangjun Da

**Affiliations:** ^1^ Shanghai Key Lab for Urban Ecology Process and Eco‐Restoration School of Ecological and Environmental Sciences East China Normal University Shanghai China; ^2^ Tiantong National Station of Forest Ecosystem Ningbo China; ^3^ Department of Conservation Biology Institute of Environmental Sciences Leiden University Leiden The Netherlands; ^4^ Center for Material Cycles and Waste Management Research National Institute for Environmental Studies Tsukuba Japan

**Keywords:** aquatic plants, facilitation, freshwater lakes, species distribution model

## Abstract

Water eutrophication creates unfavorable environmental conditions for submerged macrophytes. In these situations, biotic interactions may be particularly important for explaining and predicting the submerged macrophytes occurrence. Here, we evaluate the roles of biotic interactions in predicting spatial occurrence of submerged macrophytes in 1959 and 2009 for Dianshan Lake in eastern China, which became eutrophic since the 1980s. For the four common species occurred in 1959 and 2009, null species distribution models based on abiotic variables and full models based on both abiotic and biotic variables were developed using generalized linear model (GLM) and boosted regression trees (BRT) to determine whether the biotic variables improved the model performance. Hierarchical Bayesian‐based joint species distribution models capable of detecting paired biotic interactions were established for each species in both periods to evaluate the changes in the biotic interactions. In most of the GLM and BRT models, the full models showed better performance than the null models in predicting the species presence/absence, and the relative importance of the biotic variables in the full models increased from less than 50% in 1959 to more than 50% in 2009 for each species. Moreover, co‐occurrence correlation of each paired species interaction was higher in 2009 than that in 1959. The findings suggest biotic interactions that tend to be positive play more important roles in the spatial distribution of multispecies assemblages of macrophytes and should be included in prediction models to improve prediction accuracy when forecasting macrophytes’ distribution under eutrophication stress.

## 1 INTRODUCTION

Nutrient enrichment in aquatic systems is a widespread environmental problem caused by the massive conversion of the earth's land surface to agriculture or urban land as well as global climate change and nitrogen deposition (Jeppesen et al., 2010). As a result, regime shifts in shallow lake ecosystems have been widely documented in temperate and tropical regions (Mesters, 1995; Sand‐Jensen et al., 2008; Scheffer, Hosper, Meijer, Moss, & Jeppesen, 1993; Zhang et al., 2016). Within these aquatic ecosystems, submerged macrophytes dominate in clear oligotrophic water and phytoplankton dominates in turbid eutrophic water. In certain cases, the dominance of floating plants may represent as a third state (Mesters, 1995; Scheffer et al., 2003). In the case of both phytoplankton dominance and floating plant dominance, phytoplankton blooms or dense mats of free‐floating plants reduces the depth to which light penetrates and increases anoxic conditions, leading to an unfavorable environment for submerged macrophytes (Bornette & Puijalon, 2011). As a result, suitable habitats for submerged macrophytes are reduced and only occur near the lakeshore, even disappear in nutrient‐rich situations (Declerck et al., 2005; Kosten, Kamarainen, et al., 2009; Scheffer et al., 1993).

In unfavorable environments, the reduction in habitat can lead to intense competition among species occupying overlapping niches (Hao, Wu, Shi, Liu, & Xing, 2013; Maestre, Callaway, Valladares, & Lortie, 2009; Sand‐Jensen et al., 2008). In contrast, positive species interactions may arise as an important mechanism to maintain diversity according to the prediction of the stress‐gradient hypothesis (McIntire & Fajardo, 2014). In both cases, biotic interactions, including competition and facilitation, play an important role in explaining species coexistence. Empirical studies have revealed that macrophytes affect many key processes that promoted water clarity (Kéfi, Holmgren, & Scheffer, 2016; Scheffer et al., 1993). This vegetation–turbidity interaction ensures a low chlorophyll‐a concentration and high Secchi depths in eutrophic shallow lakes that are vegetated (Kosten, Lacerot, et al., 2009). Therefore, submerged species that are adapted to growing in nutrient‐rich, turbid water may have positive effects on species that prefer nutrient‐poor, clear water habitats, which is a type of indirect facilitation (Le Bagousse‐Pinguet, Liancourt, Gross, & Straile, 2012).

Abiotic factors are commonly used to predict the spatial distribution of submerged macrophytes: Among which transparency and depth, both related to light conditions, are often shown to be most important variables, followed by chemical contents, hydrographic parameters, substrate conditions, and other factors (Lehmann, 1998; Schmieder & Lehmann, 2004; Snickars et al., 2014; Wang et al., 2005; Zhang et al., 2016). The impacts of vertical interactions such as herbivory pressure or algae cover occasionally affect these predictions (Snickars et al., 2014; Viana et al., 2016). Few studies have considered horizontal interactions (i.e., the relation between submerged macrophytes) as explanatory factors. Recent studies have demonstrated that the incorporation of horizontal biotic interactions improves the accuracy of predictions on both a plot scale (Nylén, Le Roux, & Luoto, 2013; le Roux, Lenoir, Pellissier, Wisz, & Luoto, 2013) and a regional scale (Latimer, Banerjee, Sang, Mosher, & Silander, 2009) and have indicated that the interactions among plants play an important role in the spatial distribution of multispecies assemblages (Nylén et al., 2013; le Roux et al., 2013).

Here, we evaluated the relative importance of biotic interactions in a spatial assemblage of submerged macrophytes and how the biotic interactions between species pairs changed due to regime shifts using species distribution models. To this end, we analyzed the spatial presence/absence data of four common submerged macrophytes during two periods (1959 and 2009) in Dianshan Lake, which became eutrophic since the 1980s. We hypothesized that (H1) models with both abiotic and biotic variables would more accurately predict spatial distributions than models including only abiotic variables in each alternative regime state (Nylén et al., 2013; le Roux et al., 2013), and the improvement effects could enhance in eutrophication state. Moreover, we expected (H2) that the biotic interactions would be more positive (facilitation) due to the regime shift caused by eutrophication (Brooker et al., 2008; Le Bagousse‐Pinguet et al., 2012; McIntire & Fajardo, 2014). By testing the two hypotheses, we would provide a field evidence of how the role of biotic interaction changes in governing submerged macrophytes community assemblage before and after eutrophication.

## 2 MATERIALS AND METHODS

### 2.1 Study site

**Figure 1:**
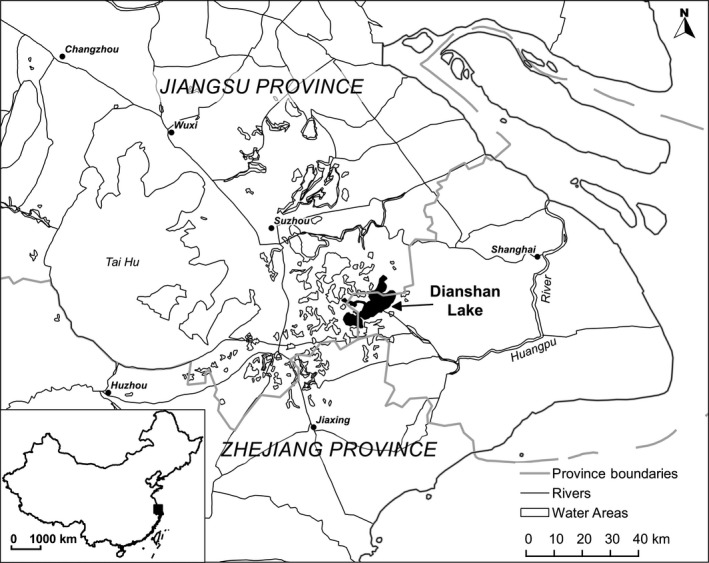
The location of Dianshan Lake, eastern China

The study was carried out in Dianshan Lake (31°04′N–31°12′N and 120°54′E–121°01′E), which is at the western margin of Shanghai and is bounded by Jiangsu and Zhejiang Provinces (Figure 1). Dianshan Lake belongs to the Taihu Lake drainage in eastern China and receives the upstream water from the Taihu Lake. Downstream, the Huangpu River is the largest river flowing through the metropolitan area of Shanghai and merges into the Yangtze River just before entering the East China Sea. The area of the lake is 63.7 km^2^, and the mean depth is ~2.1 m (Kung & Ying, 1991; Zhang, Zhang, & Zhong, 2011).

Since the early 1980s, in association with rapid economic development and urban expansion, the water quality of Dianshan Lake has deteriorated, and the lake has experienced cyanobacterial algal blooms since the end of the last century (Cheng & Li, 2010). The trophic state of Dianshan Lake transitioned from eutrophic to hypereutrophic in 1999–2000 (Cheng & Li, 2010). As a result, the aquatic ecosystem has gradually become degraded and has switched from a macrophyte‐dominated clear water system to a turbid, phytoplankton‐dominated system (Shi, Liu, & Da, 2011). The aquatic vegetation, which was present throughout the entire lake in 1959, covered no more than 30% of the lake in 2009 (Shi et al., 2011). Although the overall richness of the vascular aquatic plants has changed little, ten native species have disappeared and non‐native species have increased from 1959 to 2009 (Shi et al., 2011). In response, the fish richness has declined markedly, particularly for herbivores and piscivores (Hu et al., 2014).

### 2.2 Species distribution data

The species distribution data for the two alternative regimes were collected in 1959 and 2009. The data of 1959 were recorded during the stage of macrophytes‐dominated clear water, and the data from 2009 were recorded during the stage of phytoplankton‐dominated turbid water.

In 2009, macrophytes were surveyed during the growing season at 163 sampling sites on twenty‐two parallel transect lines oriented in the east‐west direction. The distance between transect lines was 600 m, and the distance between sampling sites was 700 m. At each sampling site, an area of ~500 m^2^ was surveyed within a 12.5‐m‐radius circle. Moreover, additional sample sites at the border of the lake where macrophytes occurred were surveyed. At each sampling site, emergent plants and floating plants were identified, and the mean height and Braun‐Blanquet cover‐abundance scale (Braun‐Blanquet, 1964) of each species were estimated. Submerged macrophytes were collected randomly 3–5 times using a grapple (0.4 m × 0.5 m) to access plants from a boat.

The 1959 data were taken from spatial species distribution maps in a historical report (Freshwater Aquaculture Lab, 1960). The historical survey aimed to document species distribution across the whole lake. The macrophyte data were collected from 55 sampling sites that were evenly distributed across the lake, and each sampling site represented an area of slightly more than 1 km^2^. In each site, macrophytes samples were randomly taken in 3–10 sampling points using the bottom trawling method. The presence/absence of macrophytes was recorded at a total of 375 sampling points.

Four species—*Potamogeton malaianus*,* Myriophyllum spicatum*,* Vallisneria natans,* and *Hydrilla verticillata*—were chosen to model the spatial distribution of submerged macrophytes because they were the dominant submerged macrophytes species during the both periods, and the other submerged macrophytes were too rare for spatial distribution modeling. For each species, the spatial point data were transferred to 117 raster cells with 30‐s resolution with area ~0.75 km^2^ and the presence/absence was determined for each cell (Fig. S1). Given that the different sampling density can cause bias in biotic interaction estimation, we compared the sampling density in area vegetated by rooted macrophytes in the two periods. The sampling point density in both periods was around four sampling sites per cell unit. Therefore, the cell‐based data were considered to be comparable between two periods as they had similar sampling density for area vegetated by rooted macrophytes (Appendix S1).

### 2.3 Environmental data

Based on previous studies, water depth (Z_M_), transparency (Z_SD_), water pH (W_pH_), dissolved oxygen (DO), total nitrogen (TN)/nitrate concentration (NO_3_^−^‐N), and total phosphorus (TP)/phosphate concentration (PO_4_^3−^‐P) were chosen as abiotic explanatory variables (Table S1). The first two variables are commonly used as proxies for light conditions and are considered to be the most important abiotic variables for submerged macrophytes (Sand‐Jensen et al., 2008; Wang et al., 2005; Zhang et al., 2016). The other chemical variables are considered to be linked to the physiology, occurrence, life‐history traits, and community dynamics of submerged macrophytes (Bornette & Puijalon, 2011; Snickars et al., 2014).

In 2009, Z_M_ and Z_SD_ were measured at 113 monitoring points. W_pH_, DO, TN, and TP were measured at 30 monitoring points. All abiotic variables were measured during the growing season of macrophytes, from May to September. For 1959, Z_SD_, W_pH_, DO, NO_3_^−^‐N and PO_4_^3−^‐P data were taken from the historical report (Freshwater Aquaculture Lab, 1960); these data were measured at 16 monitoring points from May to July. For both periods, the environment data were collected for three times. Because the lake was oligotrophic in 1959, with few organic and inorganic pollutants, NO_3_^−^‐N and PO_4_^3−^‐P were taken into account as indices of nutrients. Z_M_ was assumed to remain the same between the two periods because the mean depth of the lake varied little; it was ~2.0 m in both historical documents and in our survey results.

Z_M_ and Z_SD_ were measured with a calibrated stick and a Secchi disc, respectively. W_pH_ was measured in the field with a pHep^®^5 pH/Temperature Tester, HI 98128 (Hanna Instruments, Texas, USA) during 2009 and was determined using a colorimetric method in 1959. DO was determined using the iodometric method. NO_3_^−^‐N was determined by a spectrophotometric method using phenol disulfonic acid. PO_4_^3−^‐P was determined according to an ammonium molybdate spectrophotometric method. TP was digested with alkaline K_2_S_2_O_8_ and determined according to the ammonium molybdate spectrophotometric method. TN was digested with alkaline K_2_S_2_O_8_ and determined using the ultraviolet spectrophotometric method. All the analytical methods were following Chinese Water Analysis Methods Standards (Huang et al., 1999).

For each abiotic variable, the mean value of three collections was calculated for each sampling site. Then, the inverse distance weighting and ordinary kriging interpolation methods were applied to estimate the predicted values for the cell‐based data of species occurrence. Because there was a high correlation between the interpolated results of the inverse distance weighting method and the ordinary kriging method for each abiotic variable (*r* ranged from 0.942 to 0.988), we used only the predicted results from the inverse distance weighting method for statistical analysis.

### 2.4 Statistical analysis

#### 2.4.1 Test for hypothesis one (H1)

To test whether the presence of other submerged macrophytes (biotic variables) could improve the model predictions of species occurrence, two models were run for each species as follows: a model with only abiotic variables (null model) and a model with both abiotic and biotic variables (full model):

Full model: Occurrence_[one species]_ = abiotic variables + occurrence_[the other three species]_

Null model: Occurrence_[one species]_ = abiotic variables

Principal components analysis was applied to the abiotic variables to avoid multicollinearity, which artificially increases the amount of variation explained (Legendre & Legendre, 2012).The generated component scores were used in the models for abiotic variables. The occurrences of the other three species were used as the biotic variables for each species.

To account for potential differences related to statistic methodologies, generalized linear models (GLMs) and boosted regression trees (BRTs) were used to model variation in the occurrence of the four macrophytes (Elith, Leathwick, & Hastie, 2008; Franklin, 2009). For the GLMs, species occurrence was fitted with a binomial family and logit link function. To limit model complexity, statistical interaction terms were not included in the GLMs due to a lack of a priori knowledge of contingencies among the predictor variables. The relative importance of each predictor variable was calculated using the change in deviance after the exclusion of that variable from a GLM model containing all variables and then scaling it to 100, with higher values indicating a stronger influence on the response variable (le Roux et al., 2013).

BRTs are a machine learning method that combines the strengths of two algorithms—regression trees and boosting—with the advantages of handling different types of predictor variables with no need for a priori specification of a data model (Elith et al., 2008; Schibalski, Lehtonen, & Schröder, 2014). BRTs automatically incorporate interactions between predictors and are capable of fitting complex nonlinear relationships, which can cover the shortcomings that the GLM models were without interaction terms. The measure of variable importance in this method is based on both the frequency with which a variable is selected and the improvement resulting from the inclusion of the variable, with higher values (ranging from 0‐100) indicating a stronger influence (Elith et al., 2008; le Roux et al., 2013). All BRT models were fitted assuming a Bernoulli distribution and setting the tree complexity to 5, the learning rate to 0.005 (reduced to 0.001 for species for which the calculated trees were inadequate) and the bagging fraction to 0.75.

To quantify model performance, repeated fourfold cross‐validation was used with 999 repeats due to our small data set. For each repeat, fourfold data partitioning with random assignment was used to divide the data into a training group and a testing group for model validation. The pairwise distance sampling method was then used to remove any spatial sorting bias (Hijmans, 2012). The area under the receiver operating characteristic curve (AUC) (Fielding & Bell, 1997) was calculated for each model. The predicted occurrence probabilities were then converted to binary presence/absence predictions using a threshold that maximized the sensitivity and specificity of the model during cross‐validation, from which the true skill statistic (TSS) and kappa were calculated (Allouche, Tsoar, & Kadmon, 2006). The differences in the predictive power between null models and full models were tested using a Mann–Whitney test (Nylén et al., 2013; le Roux et al., 2013).

Because AUC values can be affected by spatial autocorrelation (Segurado, Araújo, & Kunin, 2006), all model residuals were checked for spatial autocorrelation by computing spline correlograms (Dormann et al., 2007; Schibalski, Lehtonen, & Schröder, 2014). Except slight spatial autocorrelation occurred in the BRT models of *Hydrilla verticillata* in 2009 and of *Myriophyllum spicatum* in 1959, no residual spatial autocorrelation was found.

#### 2.4.2 Test for hypothesis two (H2)

To test whether a positive shift (facilitation) of biotic interactions could arise among submerged macrophytes due to eutrophication, the co‐occurrence pattern of the four submerged macrophyte species was fitted using joint species distribution models for both periods (1959 and 2009) (Pollock et al., 2014). This method is distinct from the species distribution model, which only includes the unidirectional influence of one or a few species; using error matrices (i.e., Σ in eqn(1)) in hierarchical multivariate probit regression models allows species interactions involving multiple species to be modeled explicitly using spatial co‐occurrence data (Kissling et al., 2012). In addition, this joint modeling method has ability to decompose the species coexistence into shared environments caused coexistence and biotic interaction caused coexistence (Pollock et al., 2014).

In each model, the response is the species occurrence matrix (with element *Y*_*ij*_) arranged as *n* sites by *m* species. The probability of occurrence was defined as the probability density of a latent variable (*Z*_*ij*_) greater than zero (eqn. (1)). The row vectors of the latent variable matrix, *Z*_*i*_, follow an *m*‐dimensional multivariate normal distribution with a mean vector $\mu_{i}$ and an *m *× *m* variance/covariance matrix Σ.

(1) $$\begin{array}{cc} & \text{Pr}\left(Y_{ij}=1\right)=\text{Pr}\left(Z_{ij}>0\right),\text{for}\;i=1,\ldots ,\;n;\;j=1,\ldots ,\;m\\ & Z_{i}∼N_{j}\left(\mu_{i},\Sigma \right)\\ & \mu_{i}=X_{i}\beta \\ & \beta ∼N\left(\mu_{k},\;\sigma_{k}\right) \end{array}$$

$\mu_{i}$ is predicted with an environmental data matrix (*X*) with the dimensions *n* sites by *K* predictors, which included one column of intercept terms set as one and *K *− 1 columns of principal components of biotic variables that were centered on zero and scaled by their standard deviations. β is the m×*K* matrix of regression coefficients, which were drawn from the normal distribution with mean $\mu_{k}$ and standard deviation $\sigma_{k}$ for each column.

The inverse Wishart distribution was used as the prior for the variance/covariance matrix Σ. After model fits, the covariance terms were divided by the standard deviations of the variance/covariance matrix (Σ) to generate a correlation matrix in which all standard deviations were equal to one. The element in the rescaled variance/covariance matrix presents pairwise correlation coefficient between two species, an indicator of biotic interaction (Pollock et al., 2014).

To fit the models, sampling from the posterior distribution of all parameters was performed using the Markov chain Monte Carlo method. For each model, 1,000,000 iterations of three independent Markov chains were run, in which a total of 1,000 values were sampled with 980 iteration intervals after a burn‐in of 20,000 iterations. The priors for all model parameters used the same settings as used by Pollock et al. (2014). Gelman and Rubin diagnostics (Gelman, Carlin, Stern, & Rubin, 2003) were used to check the convergence of the Markov chains for each parameter, and if the Gelman‐Rubin statistic (potential scale reduction factor) was smaller than 1.1, the chains were considered to have converged.

The positive or negative interaction for each species pair was considered significant if the 95% interval of the distribution of the corresponding coefficient in the rescaled variance/covariance matrix did not include zero. We then employed a Mann–Whitney test to determine whether there was a significant shift in biotic interactions between two periods for each pair of species. For each species pair, when the coefficient in 2009 was significantly bigger than that in 1959, it was considered that a positive shift occurred after eutrophication. In addition, the species similarity based on seven plant traits was calculated (Pan, Zhang, Song, & Da, 2017; see Appendix S2 for details) and was compared between two groups of species pairs (see results for details).

Moreover, to determine whether biotic variables within trophic levels affected the coefficient of biotic interaction, chlorophyll‐a content (as a proxy of algal abundance), richness of emergent macrophytes, and richness of free‐floating macrophytes were included in a new hierarchical model for 2009. By comparing the results of two hierarchical models for 2009, the coefficient biotic interaction had little change by adding those biotic factors into hierarchical model (Fig. S2).

All analyses were conducted in R.3.2.2 (R Core Team, 2015); we used “gstat” package (version 1.1‐3) to interpolate environmental variables, and “gbm” package (version 2.1.1) and “dismo” package (version 1.0‐12) for BRT models fit, and “ncf” package (version 1.1‐5) to compute spline correlograms. JAGS (version 3.4.0) was run through package “R2jags” (version 0.05‐01) to fit the joint species distribution models using the code of Pollock et al. (2014).

## 3 RESULTS

In most cases, the full models with both abiotic and biotic variables performed better than the null models only with abiotic variables (Table 1) in predicting species presence/absence for the four submerged macrophytes, no matter whether the GLM or BRT method was used. For *Hydrilla verticillata* and *Vallisneria natans*, the performances of the full models were similar to those of null models for 1959 but were clearly better than those of the null models for 2009. Moreover, for each species, both the GLM and BRT models for 2009 performed better than those for 1959.

**Table 1:** Comparison of model performance for the null model (only with abiotic variables) and the full model (with both abiotic and biotic variables) for generalized linear models and boosted regression trees, respectively. Area under the receiver operating characteristic curve (AUC), true skill statistic (TSS), and kappa were used to measure model performance. The significant differences in model performance between null models and full models (Full‐Null) were tested by a Mann–Whitney test and are shown in bold type. Hydrvert: *Hydrilla verticillata*; Myrispic: *Myriophyllum spicatum*; Potamala: *Potamogeton malaianus*; Vallnata: *Vallisneria natans*

Model	Generalized linear models						Boosted regression trees					
	1959			2009			1959			2009		
	Null	Full	Full‐Null	Null	Full	Full‐Null	Null	Full	Full‐Null	Null	Full	Full‐Null
Hydrvert												
AUC	0.69	0.69	0.00	0.75	0.82	**0.06**	0.64	0.65	0.00	0.78	0.84	**0.06**
TSS	0.63	0.61	−0.01	0.78	0.96	**0.18**	0.69	0.60	−0.09	0.84	0.89	**0.06**
kappa	0.49	0.49	0.00	0.60	0.72	**0.12**	0.45	0.45	0.00	0.65	0.73	**0.08**
Myrispic												
AUC	0.66	0.76	**0.09**	0.80	0.86	**0.06**	0.62	0.70	**0.08**	0.77	0.84	**0.08**
TSS	0.65	0.57	−0.08	0.77	0.94	**0.17**	0.60	0.58	−0.01	0.86	0.89	**0.03**
kappa	0.44	0.57	**0.13**	0.66	0.76	**0.10**	0.39	0.48	**0.09**	0.61	0.73	**0.11**
Potamala												
AUC	0.65	0.73	**0.08**	0.76	0.86	**0.10**	0.62	0.64	**0.02**	0.76	0.85	**0.09**
TSS	0.59	0.72	**0.13**	0.64	0.67	**0.02**	0.53	0.62	**0.09**	0.63	0.68	0.05
kappa	0.47	0.55	**0.08**	0.58	0.73	**0.15**	0.42	0.45	**0.03**	0.61	0.70	**0.10**
Vallnata												
AUC	0.54	0.55	**0.01**	0.70	0.84	**0.14**	0.58	0.58	0.00	0.70	0.83	**0.13**
TSS	0.67	0.69	0.02	0.69	0.89	**0.20**	0.44	0.45	0.01	0.73	0.90	**0.17**
kappa	0.30	0.32	**0.02**	0.55	0.72	**0.16**	0.33	0.33	0.00	0.56	0.70	**0.14**

**Figure 2:**
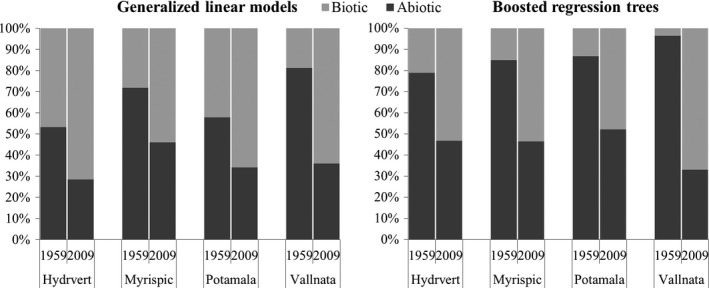
Change in relative importance of biotic variables and abiotic variables in full models between 1959 and 2009 for generalized linear models and boosted regression trees. Hydrvert: *Hydrilla verticillata*; Myrispic: *Myriophyllum spicatum*; Potamala: *Potamogeton malaianus*; Vallnata: *Vallisneria natans*

For each species, the relative importance of abiotic variables in the full models was greater than 50% for 1959 and decreased in the full models for 2009 for both GLM and BRT approaches (Figure 2). In contrast, the biotic variables increased their relative importance to more than 50% (except *Potamogeton malaianus*, with a relative importance of 47.5%) in the full models for 2009 compared with the full models for 1959.

**Figure 3:**
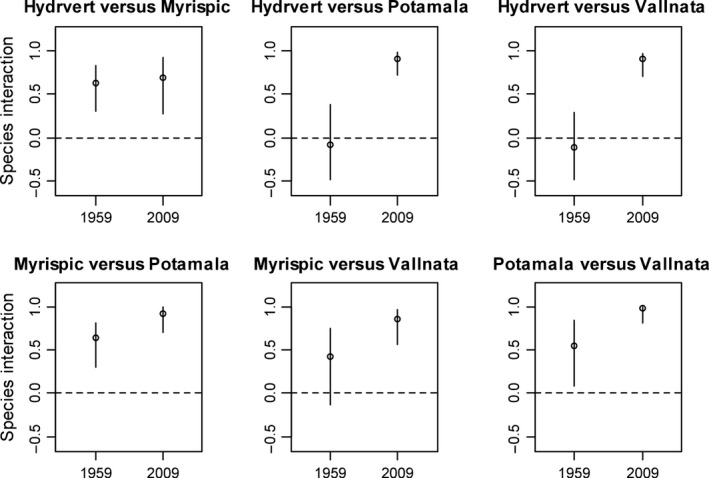
The difference in pairwise species interactions between 1959 and 2009 was determined by using the posterior distribution of coefficients in the rescaled variance/covariance matrix. Interaction coefficients are shown as the median (open circle) with 95% confidence intervals. The result was considered a significant positive or negative interaction if the 95% confidence interval did not include zero (dotted line). For each pair, there was a significant increase in the interaction coefficient from 1959 to 2009 at the 0.001 level, as tested by a Mann–Whitney test. Hydrvert: *Hydrilla verticillata*; Myrispic: *Myriophyllum spicatum*; Potamala: *Potamogeton malaianus*; Vallnata: *Vallisneria natans*

In the joint species distribution models, rescaled variance/covariance matrix (rescaled Σ) showed the residual correlation between species after controlling environmental correlation (species correlation due to their shared environmental responses). The shift of biotic interaction after eutrophication can be inferred by comparing the pairwise species residual correlation in 1959 and 2009 (Figure 3). Among the six species pairs, *Hydrilla verticillata* versus *Potamogeton malaianus*,* Hydrilla verticillata* vs. *Vallisneria natans*, and *Myriophyllum spicatum* versus *Vallisneria natans* showed neutral interaction in 1959 as the 95% confidence intervals of their coefficients in rescaled Σ include zero. In 2009, the interaction of the three species pairs became positive as theirs coefficients in rescales Σ were significantly bigger than zero at 95% confidence level and thus performed a positive shift pattern from a neutral interaction to a positive interaction after eutrophication. For the other three species pairs, the positive interaction became more intense significantly. In summary, all of the biotic interactions among species showed significant positive shifts from 1959 to 2009 (Figure 3). Furthermore, the three species pairs that showed a shift from neutral interaction to positive interaction had a significantly higher trait dissimilarity than the other three species pairs (one‐way ANOVA, *df* = 5, *p* < 0.05) (Appendix S2). Adding biotic variables within trophic levels, such as chlorophyll‐a content (as a proxy of algal abundance), richness of emergent macrophytes, and richness of free‐floating macrophytes, in the hierarchical model of 2009, did not change the high positive biotic interactions among the four submerged macrophytes (Fig. S2).

## 4 DISCUSSION

Changes in biotic interactions have been widely recorded during global changes, particularly biological interactions in response to climatic change (Brooker et al., 2008; Tylianakis, Didham, Bascompte, & Wardle, 2008). In addition to global warming's impacts on aquatic vegetation (Alahuhta, Heino, & Luoto, 2011; Kosten, Kamarainen, et al., 2009; Netten, Van Zuidam, Kosten, & Peeters, 2011), eutrophication is another, and perhaps more serious, global problem for inland waters (Davidson et al., 2015). Although there are a few controlled experiments demonstrating changes in the biotic interactions of aquatic plants in response to different levels of eutrophication (Hao et al., 2013; Le Bagousse‐Pinguet et al., 2012), to our knowledge there are few field reports describing how the interactions among aquatic vegetation respond to the freshwater ecosystem regime shift caused by eutrophication. In this study, using the species distribution models, we demonstrated that biotic interactions improved model performance and showed relative dominant roles in explanation of species occurrence comparing to abiotic factors after eutrophication. Moreover, biotic interactions showed a positive shift from a stage of oligotrophic clear water to a stage of eutrophic turbid water. This result confirmed that the positive interactions recorded under eutrophication in control experiments could also occur in the field for submerged macrophytes, in support of the stress‐gradient hypothesis (Maestre et al., 2009).

The limited light conditions associated with decreased transparency were probably the major driver of the positive shift in species interactions. The average of transparency (Secchi depth) in Dianshan Lake during the growing season was ~0.5 m in 2009 and decreased at a rate of ~0.05 m per year after 2000 (Cheng & Li, 2010). Based on an empirical study, the maximum depth should be <1.8 times the Secchi depth to ensure a net biomass accumulation during the growth season in Yangtze lakes (Wang et al., 2005). That value translates to 0.9 m, which is much shallower than the mean depth (2.1 m) of this lake. As a result, the stress of low light intensity caused the submerged macrophytes to squeeze together in the shallow water along lakeshore. In this situation, it has been suggested that indirect facilitation by submerged macrophytes, which maintain clear water conditions by competing with phytoplankton for light and nutrients, is the underlying mechanism promoting species coexistence (Le Bagousse‐Pinguet et al., 2012). This is supported by the evidence that the mean value of transparency was significantly higher in macrophytes beds (0.55 m) than outside beds (0.46 m) in 2009 (*t* test, *p* < 0.001). Under indirect facilitation, an increased richness of submerged macrophytes could lead to higher macrophytes biomass and greater chance of including high‐periphyton‐production macrophytes and then increase the biomass of periphyton and retain greater quantities of polluting nutrients such as phosphorus (Engelhardt & Ritchie, 2001), which in turn further ameliorate eutrophic stress.

Based on empirical documentation of different plant traits among benefactor and beneficiary species (Beltrán, Valiente‐Banuet, & Verdú, 2012), submerged macrophytes with greater trait divergence may exhibit more intensive facilitation. Reduced niche overlap might alleviate competition in a crowded space and increase the probability of coexistence, which in turn may enhance the indirect facilitation. This pattern was confirmed by two lines of evidence in this study. The first one is the concordance between the increase in positive interactions from 1959 to 2009 and trait divergence among the four submerged macrophytes (Appendix S2). The second line of evidence is that *Hydrilla verticillata* and *Vallisneria natans*, with greater trait differences compared with the other species, showed significantly improved model performance when biotic variables were added (Table 1). Our findings are consistent with the suggestion that trait divergence can switch the competition‐facilitation balance (Beltrán et al., 2012) and conversely imply that facilitation increases trait dispersion at the community level (McIntire & Fajardo, 2014).

Many studies have concluded that biotic interactions have an important role in improving species distribution predictions (Araújo & Luoto, 2007; Kissling et al., 2012; Nylén et al., 2013; le Roux et al., 2013); our results support this conclusion for macrophytes, particularly in the case of eutrophication that increased indirect facilitation among macrophytes. This finding suggests that the biotic interaction among macrophytes could be critical in predicting their occurrence. Moreover, our study also suggests that integrating biotic interactions into species distribution models can promote the prediction accuracy even beyond the community scale. In contrast to experiments at a fine grain of no more than several square meters (Hao et al., 2013; Le Bagousse‐Pinguet et al., 2012), our results were based on a relatively larger grain of approximately 0.75 km^2^. Both of simulation study (Araújo & Rozenfeld, 2014) and empirical analysis (Belmaker et al., 2015) indicated that positive interactions may decrease with increase in grains but can remain important even at coarse grains.

In this study, the application of joint species distribution models is novel to aquatic system, which provides a feasible way to explore how environment changes (i.e., eutrophication) affect biotic interactions for aquatic plants besides controlled experiment. However, modeling results must be carefully explained due to common limitations. First, the missing environmental covariates could affect biotic interactions inference (Kissling et al., 2012; Pollock et al., 2014). Although major environmental factors for submerged macrophytes in the shallow water of eastern China has been covered in the models (Wang et al., 2005; Zhang et al., 2016), other important abiotic factors, such as sediment characteristics and hydrometeorology variables, could change the distribution of macrophytes. And the different nutrients indices used in two periods could also bring bias. Second, biotic interaction among trophic levels, that is, herbivory stress(Hu et al., 2014), may also alter species interaction, in spite of the fact that other biotic factors, that is, algae, emergent macrophytes, free‐floating macrophytes, had few effects on inference of interaction coefficients (Fig. S2). Thirdly, we only used one growth season data in each period nearly 50 years apart, which might ignore the effort of historical accidental events, such as human disturbances (Shi et al., 2011), and did not consider seasonal variation of macrophytes composition and environment factors. Therefore, this modeling method should be applied widely to different aquatic ecosystem with long‐term observation data to further verify its performance.

Additionally, there are two key questions that must be addressed to improve the modeling of biotic interactions in multispecies assemblages of macrophytes. First, as with many joint species distribution models (Leach, Montgomery, & Reid, 2016; Pollock et al., 2014; Wisz et al., 2013), the pairwise interactions were assumed to have the same interaction type and strength in our study due to little prior knowledge. However, interactions may be different in nature and asymmetric in strength between two submerged macrophytes. For example, Hao et al. (2013) noted that the presence of *Potamogeton macckianus* facilitated the growth of *Myriophyllum spicatum* under eutrophication (total nitrogen 4.0 mg/L and total phosphorous 1.0 mg/L), whereas the presence of *M. spicatum* inhibited the growth of *P. macckianus*. Moreover, the strength of the interaction may be dependent on the species’ relative abundances. In the same control experiment (Hao et al., 2013), the facilitation effect was stronger when the ratio of biomass density between *M. spicatum* and *P. macckianus* was 1:3, rather than 2:2 or 3:1. Second, changes in the strength and type of biotic interactions among macrophytes in different alterative ecosystem states highlight the risk of including biotic interaction factors in the prediction of species distribution. This challenge confirms that problems in the transferability of species distribution models may not only be caused by environmental heterogeneity (Schibalski, Lehtonen, & Schröder, 2014) but also by variable biotic interactions over space and time (Kissling et al., 2012; Tylianakis et al., 2008; Wisz et al., 2013).

To untangle these Gordian knots, we must move toward a greater integration of observed natural environmental gradients and multifactorial controlled experiments to learn how biotic interactions among macrophytes respond to eutrophication and its interaction with climate change (Jeppesen et al., 2010) and to integrate the findings into species distribution models with spatially explicit abundance data (Wisz et al., 2013). To avoid the complexity of pairwise interactions, this prior knowledge learning process should pay particular attention to communities composed of a small number of species interacting strongly (Gilman et al., 2010), which can be inferred based on plant trait similarities. As we have shown, species with greater trait divergence exhibit a greater change in the type and strength of biotic interactions and can be considered as community module worthy of further study.

## 5 CONCLUSION

The study demonstrated, including biotic interactions in modeling species distributions, can increase the prediction accuracy of macrophytes multispecies assemblages, and the role of biotic interactions in determining species distribution can be even more important than abiotic factors for macrophytes, in the case of eutrophication. Species association between macrophytes in stressful environmental, that is, low light condition in eutrophic water, become more tightly and probably tend to be positive, which has large contributions on predicting distribution of multispecies assemblages. We suggest the approaches that join horizontal biotic factors into species distribution model can be applied to a wide range of aquatic ecosystem from community scale to regional scale, which is helpful for restoration and management of eutrophic water. However, the join species distribution models built on different alterative ecosystem states cannot simply be used for each other, without extrapolating the distribution of all potentially interacting species and the variation of their interactions.

## AUTHOR'S CONTRIBUTION

SK, DA LJ designed the study; C YC, Xu JL,Z XJ, Pan YJ collected species distribution and environmental data; SK, DA LJ, Xu KQ performed data analysis and led the writing; all authors contributed to writing the manuscript.

## CONFLICT OF INTEREST

None declared.
