# Supplementary material for: Enhanced effects of biotic interactions on predicting multispecies spatial distribution of submerged macrophytes after eutrophication
Source: Ecol Evol. 2017 Aug 22;7(19):7719–28. doi: 10.1002/ece3.3294 (PMC5632620; doi:10.1002/ece3.3294)
Supplement: Supplementary file 1 [file ECE3-7-7719-s001.docx]

**Table S1.** The mean value and range for each environmental variable of the Dianshan Lake in 1959 and 2009. In 2009, transparency was measured in 113 monitoring points and the others were measured in 30 monitoring points during the growing season of macrophytes from May to September. In 1959, all variables were measured in 16 monitoring points from May to July.

| Environmental variables | 1959 | 2009 |
| --- | --- | --- |
| Transparency(m) | 0.77[0.32,1.90] | 0.52[0.22,1.05] |
| Water pH | 7.87[7.67,8.33] | 8.46[6.38,10.03] |
| Dissolved Oxygen(mg/L) | 7.57[6.89,8.17] | 7.63[4.01,11.70] |
| Nitrate(mg/L) | 0.029[0.010,0.077] | / |
| Phosphate(mg/L) | 0.005[0.002*,0.024] | / |
| Total nitrogen(mg/L) | / | 3.30[1.65,7.62] |
| Total phosphorus(mg/L) | / | 0.43[0.27,1.04] |
| chlorophyll a** |  | 27.12[0.78,284.63] |

Note: * the value is lower than detective limit of chemical analysis method used in 1959.

** measured by spectrophotometric method following Chinese Water Analysis Methods Standards（Huang *et al*., 1999）.


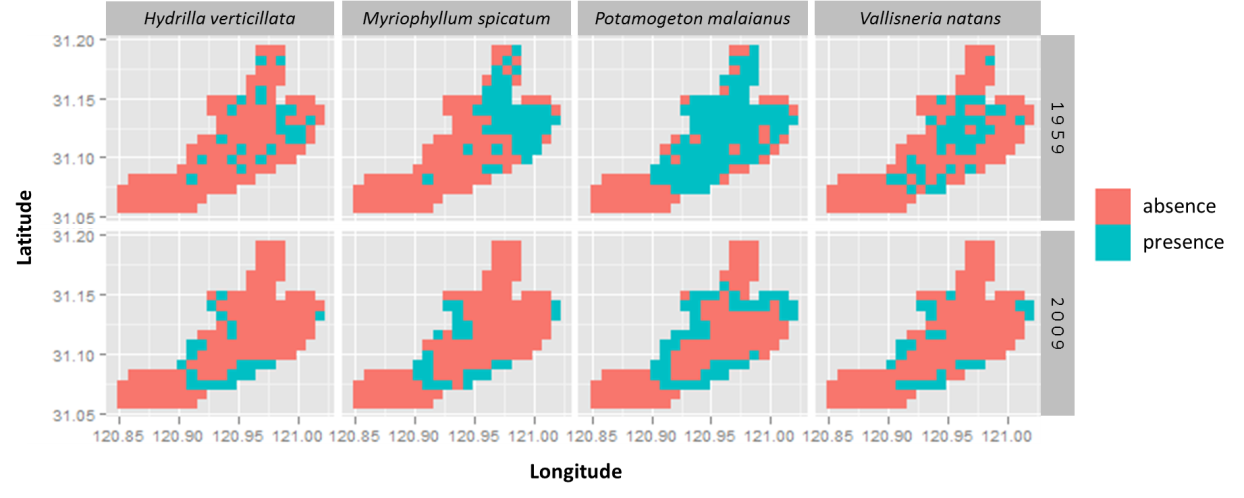


**Figure S1.** The spatial distribution of four dominated submerged macrophytes in 1959 and 2009, in the Dianshan Lake (31°04’N-31°12’N and 120°54’E-121°01’E) which is at the western margin of Shanghai.


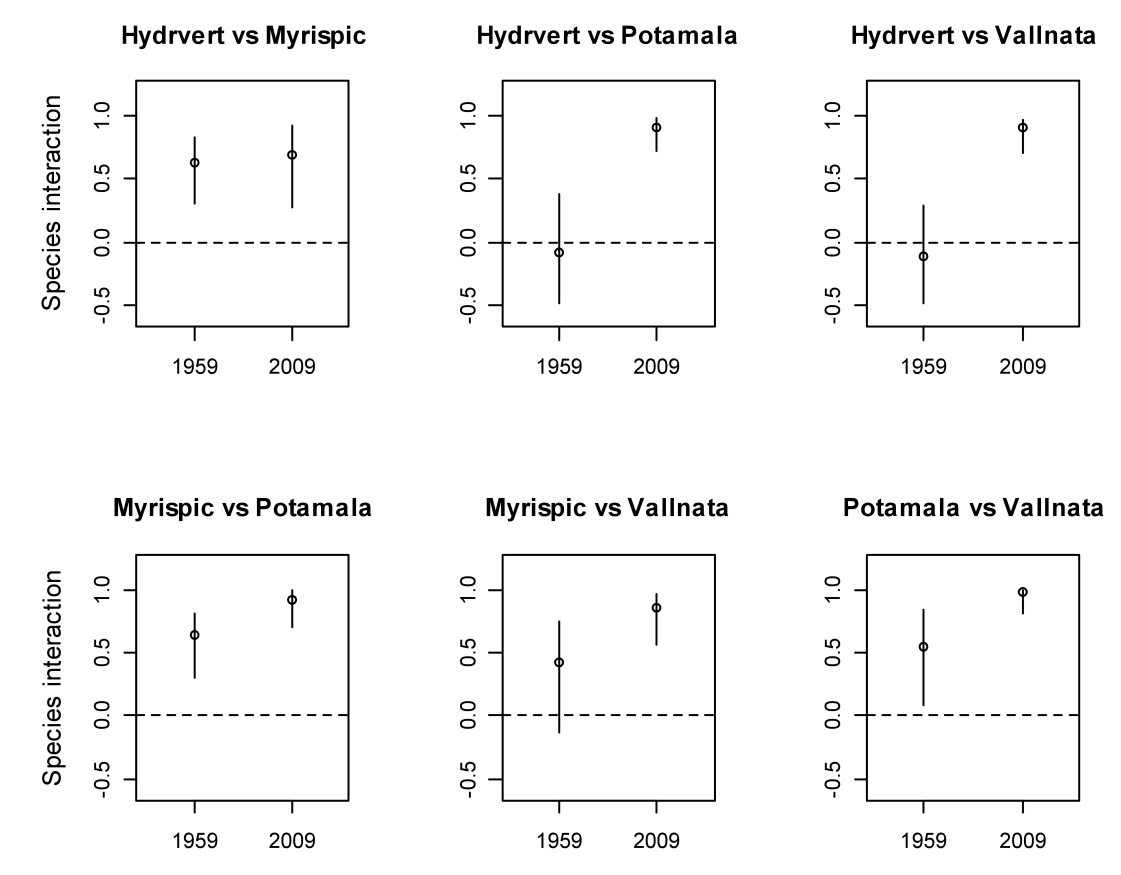


**Figure S2.** We compared species interaction based on hierarchical models including (2009new) and excluding (2009) chlorophyll-a content (as a proxy of algal abundance), richness of emergent macrophytes, and richness of free-floating macrophytes as biotic interactions within trophic levels for 2009. New interaction coefficients of 2009 became smaller than original ones in three species pairs and were almost the same in the other three species pairs. Each of them was still higher than that of 1959 and the positive shift tendency from 1959 to 2009 did not change. Interaction coefficients are shown as median (open circle) with 95% confidence intervals. The result was considered to be a significant positive or negative interaction if the 95% confidence intervals did not include zero (dotted line). Hydryert: *Hydrilla verticillata*, Myrispic: *Myriophyllum spicatum*, Potamala: *Potamogeton malaianus*, Vallnata: *Vallisneria natans*.

**Appendix S1** Comparison of sampling method in 1959 and 2009.

Different sampling methods used in 1959 and 2009 made different total sampling points across the whole lake. That is 375 sampling points in 1959 and 226 points in 2009. Given that the different sampling density can lead bias to estimate the biotic interaction, i.e. higher sampling density results higher co-occurrence possibility; we compared the sampling density in area vegetated by rooted macrophytes in the two periods. It is because changes in sampling density in area without root macrophytes would not lead estimation bias of co-occurrence possibility for the four studied submerged macrophytes. Therefore, we roughly calculated the sampling density based on the rooted macrophytes coverage (sum of areas of yellow cells inside the lake boundary, Figure S3).

The sampling point density is 4.38 per cell unit in 1959 and 4.09 per cell unit in 2009. Therefore, we consider that the sampling methods used in two periods are comparable by using cell-based data for statistical analysis.

(b)

(a)

Figure S3 Field sampling points of macrophytes in 1959 (a) and 2009 (b). Red open circles are supplement sampling points besides regular sampling points (black open circles) on twenty-two parallel transect lines in 2009. The lake bounded by solid line was divided into 117 cells with 30 second resolution. The areas vegetated by rooted macrophytes are in yellow cells and areas not vegetated by rooted macrophytes are in grey cells.

**Appendix S2** Principal coordinates ordination analysis on seven plant traits.

Principal coordinates ordination analysis (PCoA) was used to shown traits divergence among four submerged macrophytes (Figure S4), based on the Euclidean distance of seven plant traits (Table S2). According to leaf biomass distribution in depth based on field measurement in 2 - 6 plots for each species (unpublished data) (Figure S5), four species were grouped into three types: Top type – most leaf biomass aggregated at the top of plant near water surface, Bottom type - most leaf biomass aggregated at the bottom of plant near lake substrate, Top-mid type – most leaf biomass aggregated at the top to middle part of plant. BRP: root biomass percentage, SLA: specific leaf area, LDMC: leaf dry matter content, LBPa: leaf biomass percentage of aboveground biomass, Nmass: leaf nitrogen content, Pmass: leaf phosphorus content. SLA was cited from Fu et al. (2014) and RBP was measured in the early 1990s by You (1993). All the other traits were measured in 2009 (Pan et al., 2017). Hydryert: *Hydrilla verticillata*, Myrispic: *Myriophyllum spicatum*, Potamala: *Potamogeton malaianus*, Vallnata: *Vallisneria natans*


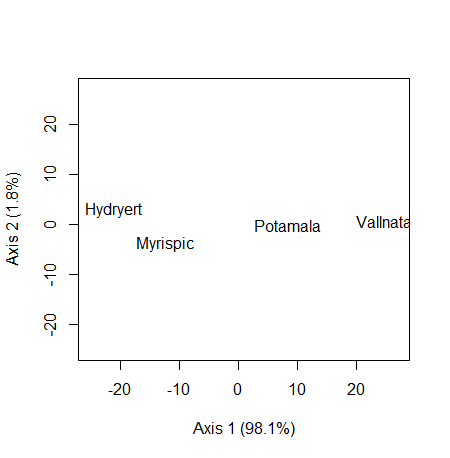


Figure S4 PCoA of four studies macrophytes based on plant traits

Table S2 Seven plant traits of four studies macrophytes


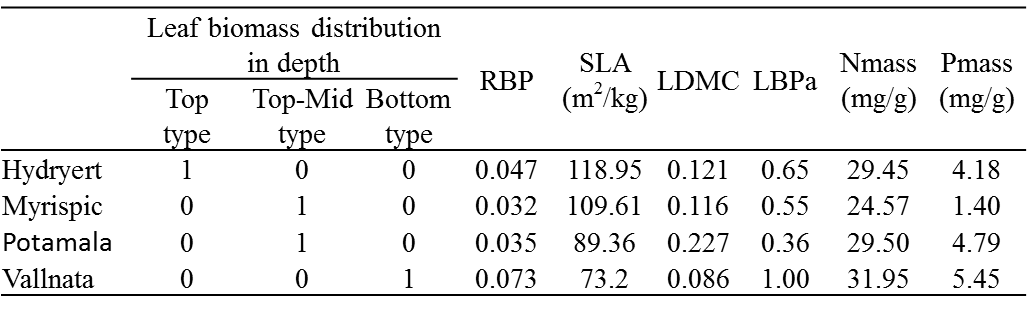


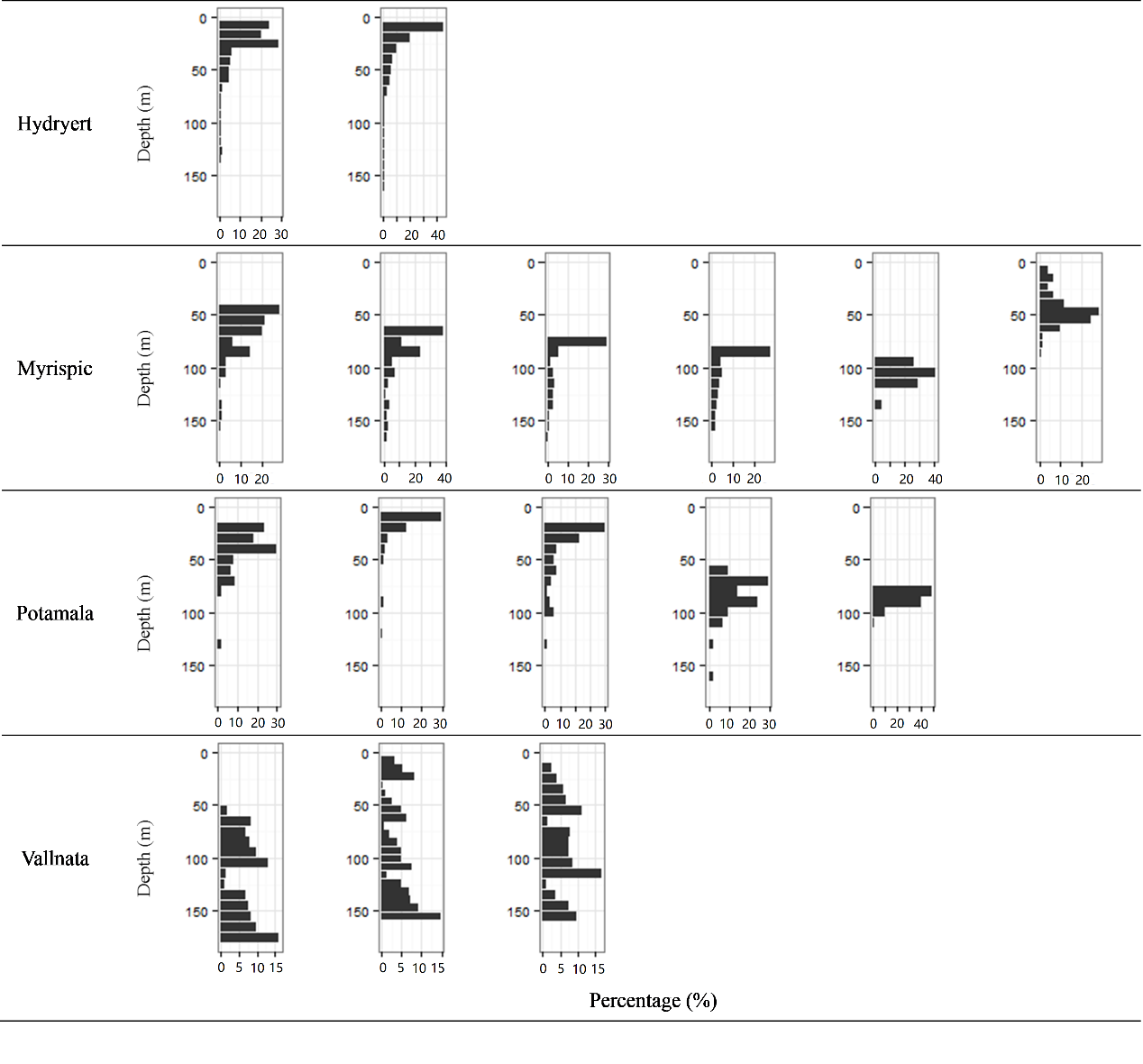


Figure S5 leaf biomass distribution in depth of four studies macrophytes in 2009

Reference:

Fu H, Zhong J, Yuan G *et al*. (2014) Trait-based community assembly of aquatic macrophytes along a water depth gradient in a freshwater lake. Freshwater Biology, **59**, 2462-2471

Pan YJ, Zhang XJ, Song K *et al*. (2017) Applying trait-based method to investigate the relationship between macrophyte communities and environmental conditions in a eutrophic freshwater lake, China. Aquatic Botany, **142**, 16-24

You WH (1993) A study on the aquatic biocoenology in the Dianshan Lake. Ph.D. thesis. East China Normal University, Shanghai.
